# Di-μ_4_-succinato-tetra­kis[aqua­phenanthrolinecopper(II)] tetra­nitrate tetra­hydrate

**DOI:** 10.1107/S1600536809039580

**Published:** 2009-10-03

**Authors:** Panana Kitiphaisalnont, Sutatip Siripaisarnpipat, Narongsak Chaichit

**Affiliations:** a Department of Chemistry, Kasetsart University, Bangkok 10903, Thailand; b Department of Physics, Thammasat University, Rangsit, Pathumthani 12121, Thailand

## Abstract

In the title compound, [Cu_4_(C_4_H_4_O_4_)_2_(C_12_H_8_N_2_)_4_(H_2_O)_4_](NO_3_)_4_·4H_2_O, the complete tetra­cation is generated by crystallographic inversion symmetry. Both unique Cu^2+^ ions are coordinated by an *N*,*N*′-bidentate phenanthroline mol­ecule, two *O*-monodentate bis-bridging succinate dianions and a water mol­ecule, resulting in distorted CuN_2_O_3_ square-based pyramidal geometries for the metal ions, with the water mol­ecule occupying the apical site. In the crystal, the components are linked by O—H⋯O hydrogen bonds and aromatic π–π stacking inter­actions [minimum centroid–centroid separation = 3.537 (2) Å].

## Related literature

For related structures, see: McCann *et al.* (1998 ▶); Padmanabhan *et al.* (2005 ▶); Ghosh *et al.* (2007 ▶).
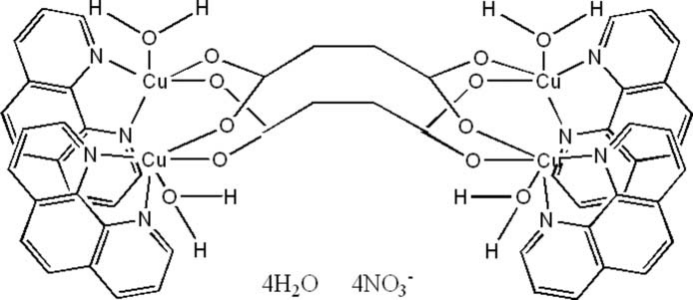


## Experimental

### 

#### Crystal data

[Cu_4_(C_4_H_4_O_4_)_2_(C_12_H_8_N_2_)_4_(H_2_O)_4_](NO_3_)_4_·4H_2_O
*M* *_r_* = 1599.29
Monoclinic, 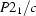

*a* = 8.9180 (1) Å
*b* = 34.1090 (2) Å
*c* = 10.3620 (2) Å
β = 96.031 (1)°
*V* = 3134.51 (7) Å^3^
*Z* = 2
Mo *K*α radiation
μ = 1.44 mm^−1^
*T* = 293 K
0.20 × 0.19 × 0.10 mm

#### Data collection

Bruker SMART 1K CCD diffractometer
Absorption correction: none
23089 measured reflections
8980 independent reflections
7772 reflections with *I* > 2σ(*I*)
*R*_int_ = 0.018

#### Refinement

*R*[*F* ^2^ > 2σ(*F* ^2^)] = 0.064
*wR*(*F* ^2^) = 0.191
*S* = 1.05
8980 reflections
463 parameters
101 restraints
H atoms treated by a mixture of independent and constrained refinement
Δρ_max_ = 2.88 e Å^−3^
Δρ_min_ = −1.59 e Å^−3^

### 

Data collection: *SMART* (Bruker, 2000 ▶); cell refinement: *SAINT* (Bruker, 2000 ▶); data reduction: *SAINT*; program(s) used to solve structure: *SHELXS97* (Sheldrick, 2008 ▶); program(s) used to refine structure: *SHELXL97* (Sheldrick, 2008 ▶); molecular graphics: *SHELXTL* (Sheldrick, 2008 ▶); software used to prepare material for publication: *SHELXTL*.

## Supplementary Material

Crystal structure: contains datablocks I, global. DOI: 10.1107/S1600536809039580/hb5112sup1.cif

Structure factors: contains datablocks I. DOI: 10.1107/S1600536809039580/hb5112Isup2.hkl

Additional supplementary materials:  crystallographic information; 3D view; checkCIF report

## Figures and Tables

**Table 1:** Selected bond lengths (Å)

Cu1—O2	1.948 (3)
Cu1—O5	1.966 (3)
Cu1—N2	2.011 (3)
Cu1—N1	2.015 (3)
Cu1—O4	2.240 (3)
Cu2—O1	1.946 (3)
Cu2—O6	1.952 (3)
Cu2—N4	2.007 (3)
Cu2—N3	2.025 (3)
Cu2—O3	2.160 (3)

**Table 2:** Hydrogen-bond geometry (Å, °)

*D*—H⋯*A*	*D*—H	H⋯*A*	*D*⋯*A*	*D*—H⋯*A*
O3—H4⋯O14	0.73 (7)	1.99 (7)	2.707 (7)	169 (7)
O3—H16⋯O13	0.70 (6)	2.07 (6)	2.772 (6)	177 (9)
O4—H23⋯O12	0.82 (6)	2.27 (6)	3.015 (7)	153 (5)
O4—H24⋯O10^i^	0.82 (5)	2.03 (6)	2.816 (6)	161 (8)
O13—H13*C*⋯O5^ii^	0.77 (7)	2.24 (7)	3.000 (5)	167 (7)
O13—H13*D*⋯O10^iii^	0.71 (7)	2.23 (7)	2.899 (7)	159 (8)
O14—H14*B*⋯O9^iv^	0.83 (7)	2.15 (6)	2.846 (13)	142 (6)
O14—H14*C*⋯O7^v^	0.82 (9)	2.10 (9)	2.874 (14)	158 (10)

Symmetry codes: (i) 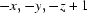; (ii) 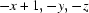; (iii) 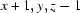; (iv) 
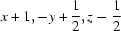
; (v) 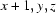.

**Fig. 1.**
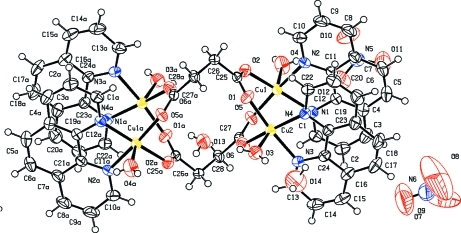
The molecular structure of (I), with 50% probability displacement ellipsoids for non-H atoms.

## supplementary crystallographic information

### Comment

The moleculular structure of the title compound, (I), consists of a tetranuclear [Cu_4_(phen)_4_(suc)_2_(H_2_O)_4_].^4+^ species and uncoordinated water molecules and nitrate anions. Each Cu(II) ion (Table 1) exhibits a distorted square pyramidal coordination geometry through one apical water oxygen atom, two phen N atoms and two carboxylate O atoms from two succinate dianions which act as bis bridging ligands toward the Cu1 and Cu2 atoms (Fig. 1).  The Cu1···Cu2 distance is 3.0318 (4) Å.  The succinate ions also bridge two Cu(II) ions (Cu1' and Cu2'). The Cu1 and Cu2' distance separated by the bridging succinate anion is 6.396 Å. The face-to-face π-π interactions between the phenanthroline ring enhance the stability of the structure.

The apical water molecules form hydrogen bonds with nitrate O atoms (O···O distances of 2.810–2.920 Å) and uncoordinated water O atoms (O···O distances of 2.709–2.768Å): Table 2.

### Experimental

The solvothermal systhesis was carried out in telflon-lined stainless steel autoclave. A mixture of Cu(NO_3_)_2_.2H_2_O, phenantholine and succinic acid (mole ratio 1:1:1) in (H_2_O)/MeOH (2:1) was heated at 423 K for 72 h. Green slabs of (I) in a green solution were obtained.

### Refinement

All the H atoms were located in a difference map and their positions and *U*_iso_(H) value were freely refined.

### Crystal data

**Table:**

[Cu_4_(C_4_H_4_O_4_)_2_(C_12_H_8_N_2_)_4_(H_2_O)_4_](NO_3_)_4_·4H_2_O	*Z* = 2
*M**_r_* = 1599.29	*F*(000) = 1632
Monoclinic, *P*2_1_/*c*	*D*_x_ = 1.694 Mg m^−^^3^
Hall symbol: -P 2ybc	Mo *K*α radiation, λ = 0.71073 Å
*a* = 8.9180 (1) Å	Cell parameters from 23295 reflections
*b* = 34.1090 (2) Å	µ = 1.44 mm^−^^1^
*c* = 10.3620 (2) Å	*T* = 293 K
β = 96.031 (1)°	Slab, green
*V* = 3134.51 (7) Å^3^	0.20 × 0.19 × 0.10 mm

### Data collection

**Table:**

Bruker SMART 1K CCD diffractometer	7772 reflections with *I* > 2σ(*I*)
Radiation source: fine-focus sealed tube	*R*_int_ = 0.018
graphite	θ_max_ = 30.5°, θ_min_ = 1.2°
ω scans	*h* = −12→9
23089 measured reflections	*k* = −37→48
8980 independent reflections	*l* = −13→14

### Refinement

**Table:**

Refinement on *F*^2^	Primary atom site location: structure-invariant direct methods
Least-squares matrix: full	Secondary atom site location: difference Fourier map
*R*[*F*^2^ > 2σ(*F*^2^)] = 0.064	Hydrogen site location: inferred from neighbouring sites
*wR*(*F*^2^) = 0.191	H atoms treated by a mixture of independent and constrained refinement
*S* = 1.05	*w* = 1/[σ^2^(*F*_o_^2^) + (0.1023*P*)^2^ + 8.0138*P*] where *P* = (*F*_o_^2^ + 2*F*_c_^2^)/3
8980 reflections	(Δ/σ)_max_ < 0.001
463 parameters	Δρ_max_ = 2.88 e Å^−^^3^
101 restraints	Δρ_min_ = −1.59 e Å^−^^3^

### Special details

**Table:**

Geometry. All e.s.d.'s (except the e.s.d. in the dihedral angle between two l.s. planes) are estimated using the full covariance matrix. The cell e.s.d.'s are taken into account individually in the estimation of e.s.d.'s in distances, angles and torsion angles; correlations between e.s.d.'s in cell parameters are only used when they are defined by crystal symmetry. An approximate (isotropic) treatment of cell e.s.d.'s is used for estimating e.s.d.'s involving l.s. planes.
Refinement. Refinement of *F*^2^ against ALL reflections. The weighted *R*-factor *wR* and goodness of fit *S* are based on *F*^2^, conventional *R*-factors *R* are based on *F*, with *F* set to zero for negative *F*^2^. The threshold expression of *F*^2^ > σ(*F*^2^) is used only for calculating *R*-factors(gt) *etc*. and is not relevant to the choice of reflections for refinement. *R*-factors based on *F*^2^ are statistically about twice as large as those based on *F*, and *R*- factors based on ALL data will be even larger.

### Fractional atomic coordinates and isotropic or equivalent isotropic displacement parameters (Å^2^)

**Table:**

	*x*	*y*	*z*	*U*_iso_*/*U*_eq_	
Cu1	0.28697 (5)	0.045902 (12)	0.25937 (4)	0.02773 (12)	
Cu2	0.45414 (5)	0.092420 (12)	0.07025 (4)	0.02759 (12)	
O1	0.5954 (3)	0.05491 (8)	0.1565 (3)	0.0403 (6)	
O2	0.4661 (3)	0.01290 (8)	0.2689 (3)	0.0384 (6)	
O3	0.6118 (4)	0.11127 (12)	−0.0618 (3)	0.0431 (7)	
O4	0.1350 (5)	0.01289 (11)	0.3799 (4)	0.0577 (9)	
O5	0.2091 (3)	0.02148 (9)	0.0939 (3)	0.0366 (6)	
O6	0.3553 (4)	0.05135 (8)	−0.0390 (3)	0.0396 (6)	
O7	−0.1911 (12)	0.2391 (4)	0.0691 (10)	0.191 (2)	
O8	−0.1382 (12)	0.2708 (4)	0.2187 (10)	0.191 (2)	
O9	−0.3494 (12)	0.2700 (4)	0.1619 (9)	0.191 (2)	
O10	−0.0018 (6)	0.06202 (15)	0.6390 (5)	0.0804 (7)	
O11	−0.1232 (6)	0.11331 (15)	0.5928 (5)	0.0804 (7)	
O12	−0.0996 (6)	0.07141 (15)	0.4439 (5)	0.0804 (7)	
O13	0.8466 (5)	0.06449 (13)	−0.1270 (4)	0.0529 (8)	
O14	0.6181 (10)	0.18964 (17)	−0.1023 (7)	0.105 (2)	
N1	0.1424 (3)	0.09146 (9)	0.2426 (3)	0.0281 (5)	
N2	0.3661 (3)	0.07455 (9)	0.4221 (3)	0.0299 (6)	
N3	0.2987 (3)	0.13232 (9)	−0.0008 (3)	0.0313 (6)	
N4	0.5046 (3)	0.13445 (8)	0.2033 (3)	0.0285 (5)	
N5	−0.0754 (7)	0.08168 (19)	0.5574 (6)	0.0804 (7)	
N6	−0.2256 (16)	0.2581 (4)	0.1483 (12)	0.191 (2)	
C1	0.0277 (4)	0.09854 (12)	0.1527 (4)	0.0348 (7)	
H1	0.0070	0.0806	0.0856	0.042*	
C2	−0.0628 (5)	0.13195 (14)	0.1556 (5)	0.0442 (9)	
H2	−0.1422	0.1359	0.0912	0.053*	
C3	−0.0343 (5)	0.15882 (13)	0.2536 (5)	0.0441 (9)	
H3	−0.0919	0.1815	0.2547	0.053*	
C4	0.0829 (4)	0.15163 (11)	0.3524 (4)	0.0357 (7)	
C5	0.1192 (6)	0.17666 (13)	0.4627 (5)	0.0502 (11)	
H5	0.0631	0.1993	0.4711	0.060*	
C6	0.2326 (6)	0.16796 (14)	0.5539 (4)	0.0524 (11)	
H6	0.2540	0.1849	0.6237	0.063*	
C7	0.3218 (5)	0.13296 (12)	0.5464 (4)	0.0396 (8)	
C8	0.4391 (6)	0.12140 (15)	0.6398 (4)	0.0508 (11)	
H8	0.4656	0.1369	0.7124	0.061*	
C9	0.5138 (6)	0.08717 (15)	0.6231 (4)	0.0507 (11)	
H9	0.5903	0.0790	0.6852	0.061*	
C10	0.4754 (5)	0.06441 (13)	0.5126 (4)	0.0404 (8)	
H10	0.5284	0.0413	0.5021	0.049*	
C11	0.2887 (4)	0.10818 (11)	0.4393 (3)	0.0302 (6)	
C12	0.1680 (4)	0.11755 (10)	0.3420 (3)	0.0287 (6)	
C13	0.1976 (5)	0.12984 (14)	−0.1039 (4)	0.0409 (8)	
H13	0.1950	0.1075	−0.1555	0.049*	
C14	0.0944 (5)	0.16024 (16)	−0.1369 (5)	0.0503 (11)	
H14	0.0256	0.1580	−0.2105	0.060*	
C15	0.0946 (5)	0.19314 (14)	−0.0612 (5)	0.0495 (10)	
H15	0.0250	0.2131	−0.0818	0.059*	
C16	0.2019 (5)	0.19638 (12)	0.0489 (4)	0.0401 (8)	
C17	0.2115 (6)	0.22905 (12)	0.1378 (5)	0.0513 (11)	
H17	0.1425	0.2495	0.1242	0.062*	
C18	0.3176 (6)	0.23065 (12)	0.2398 (6)	0.0529 (11)	
H18	0.3224	0.2524	0.2941	0.063*	
C19	0.4237 (5)	0.19925 (11)	0.2661 (4)	0.0385 (8)	
C20	0.5367 (6)	0.19841 (13)	0.3722 (5)	0.0478 (10)	
H20	0.5482	0.2194	0.4295	0.057*	
C21	0.6289 (5)	0.16652 (14)	0.3902 (4)	0.0446 (9)	
H21	0.7046	0.1660	0.4591	0.053*	
C22	0.6097 (4)	0.13465 (12)	0.3049 (4)	0.0346 (7)	
H22	0.6723	0.1129	0.3195	0.042*	
C23	0.4138 (4)	0.16638 (10)	0.1835 (4)	0.0300 (6)	
C24	0.3028 (4)	0.16508 (10)	0.0740 (3)	0.0303 (6)	
C25	0.5830 (4)	0.02380 (10)	0.2196 (3)	0.0288 (6)	
C26	0.7229 (4)	−0.00119 (11)	0.2433 (3)	0.0307 (7)	
C27	0.2562 (4)	0.02685 (10)	−0.0151 (3)	0.0276 (6)	
C28	0.1862 (4)	0.00277 (12)	−0.1284 (4)	0.0319 (7)	
H13D	0.865 (7)	0.0669 (19)	−0.191 (7)	0.054 (19)*	
H13C	0.840 (8)	0.042 (2)	−0.128 (7)	0.07 (2)*	
H4	0.624 (7)	0.132 (2)	−0.075 (6)	0.055 (18)*	
H16	0.671 (7)	0.0999 (18)	−0.081 (6)	0.047 (16)*	
H24	0.101 (10)	−0.0089 (12)	0.393 (9)	0.11 (3)*	
H23	0.080 (6)	0.0256 (17)	0.422 (5)	0.065 (19)*	
H28B	0.173 (7)	−0.0210 (18)	−0.092 (6)	0.057 (16)*	
H26B	0.704 (7)	−0.0255 (19)	0.270 (6)	0.058 (16)*	
H26A	0.773 (6)	0.0080 (14)	0.316 (5)	0.039 (12)*	
H28A	0.098 (7)	0.0156 (19)	−0.140 (6)	0.065 (18)*	
H14B	0.669 (8)	0.200 (2)	−0.155 (6)	0.10 (3)*	
H14C	0.679 (10)	0.198 (3)	−0.044 (8)	0.09 (3)*	

### Atomic displacement parameters (Å^2^)

**Table:**

	*U*^11^	*U*^22^	*U*^33^	*U*^12^	*U*^13^	*U*^23^
Cu1	0.0279 (2)	0.0305 (2)	0.0247 (2)	0.00133 (15)	0.00241 (15)	−0.00501 (15)
Cu2	0.0291 (2)	0.0243 (2)	0.0287 (2)	0.00061 (14)	−0.00019 (15)	−0.00122 (14)
O1	0.0387 (14)	0.0304 (13)	0.0505 (16)	0.0070 (11)	−0.0019 (12)	0.0056 (11)
O2	0.0359 (13)	0.0400 (14)	0.0402 (14)	0.0100 (11)	0.0079 (11)	0.0007 (11)
O3	0.0407 (16)	0.0469 (19)	0.0435 (16)	0.0031 (14)	0.0136 (13)	0.0056 (14)
O4	0.072 (2)	0.0434 (18)	0.063 (2)	−0.0140 (17)	0.0327 (19)	−0.0013 (16)
O5	0.0366 (13)	0.0438 (15)	0.0298 (12)	−0.0025 (11)	0.0053 (10)	−0.0122 (11)
O6	0.0477 (16)	0.0338 (13)	0.0368 (14)	−0.0110 (11)	0.0018 (12)	−0.0077 (11)
O7	0.146 (4)	0.276 (7)	0.139 (4)	0.073 (4)	−0.043 (3)	−0.078 (4)
O8	0.146 (4)	0.276 (7)	0.139 (4)	0.073 (4)	−0.043 (3)	−0.078 (4)
O9	0.146 (4)	0.276 (7)	0.139 (4)	0.073 (4)	−0.043 (3)	−0.078 (4)
O10	0.0886 (18)	0.0729 (15)	0.0775 (16)	0.0048 (13)	−0.0016 (13)	−0.0012 (13)
O11	0.0886 (18)	0.0729 (15)	0.0775 (16)	0.0048 (13)	−0.0016 (13)	−0.0012 (13)
O12	0.0886 (18)	0.0729 (15)	0.0775 (16)	0.0048 (13)	−0.0016 (13)	−0.0012 (13)
O13	0.062 (2)	0.051 (2)	0.048 (2)	0.0071 (17)	0.0155 (17)	−0.0016 (16)
O14	0.169 (7)	0.059 (3)	0.089 (4)	−0.035 (4)	0.023 (5)	0.007 (3)
N1	0.0273 (13)	0.0322 (14)	0.0246 (12)	−0.0023 (10)	0.0023 (10)	−0.0017 (10)
N2	0.0325 (14)	0.0333 (14)	0.0235 (12)	−0.0018 (11)	0.0004 (11)	−0.0009 (11)
N3	0.0293 (14)	0.0331 (14)	0.0307 (14)	0.0010 (11)	−0.0004 (11)	0.0028 (11)
N4	0.0275 (13)	0.0258 (13)	0.0316 (14)	0.0000 (10)	0.0008 (11)	−0.0001 (10)
N5	0.0886 (18)	0.0729 (15)	0.0775 (16)	0.0048 (13)	−0.0016 (13)	−0.0012 (13)
N6	0.146 (4)	0.276 (7)	0.139 (4)	0.073 (4)	−0.043 (3)	−0.078 (4)
C1	0.0276 (16)	0.0421 (19)	0.0333 (17)	−0.0040 (14)	−0.0037 (13)	−0.0033 (14)
C2	0.0297 (18)	0.053 (2)	0.048 (2)	0.0044 (16)	−0.0056 (16)	0.0041 (19)
C3	0.0336 (18)	0.038 (2)	0.060 (3)	0.0081 (15)	0.0050 (17)	0.0029 (18)
C4	0.0380 (18)	0.0296 (16)	0.0399 (19)	0.0033 (14)	0.0060 (15)	−0.0033 (14)
C5	0.064 (3)	0.0328 (19)	0.055 (3)	0.0055 (19)	0.010 (2)	−0.0127 (18)
C6	0.076 (3)	0.041 (2)	0.040 (2)	0.001 (2)	0.004 (2)	−0.0183 (18)
C7	0.052 (2)	0.0382 (19)	0.0274 (16)	−0.0061 (16)	0.0000 (15)	−0.0065 (14)
C8	0.067 (3)	0.055 (3)	0.0281 (18)	−0.009 (2)	−0.0080 (18)	−0.0079 (17)
C9	0.057 (3)	0.060 (3)	0.0311 (19)	−0.001 (2)	−0.0140 (18)	0.0008 (18)
C10	0.044 (2)	0.045 (2)	0.0307 (17)	0.0021 (16)	−0.0045 (15)	0.0051 (15)
C11	0.0355 (17)	0.0324 (16)	0.0225 (14)	−0.0036 (13)	0.0025 (12)	−0.0024 (12)
C12	0.0289 (15)	0.0295 (15)	0.0279 (15)	−0.0003 (12)	0.0043 (12)	−0.0034 (12)
C13	0.0392 (19)	0.051 (2)	0.0314 (17)	0.0005 (17)	−0.0034 (15)	0.0014 (16)
C14	0.043 (2)	0.065 (3)	0.040 (2)	0.004 (2)	−0.0099 (17)	0.010 (2)
C15	0.044 (2)	0.048 (2)	0.054 (3)	0.0117 (19)	−0.0056 (19)	0.015 (2)
C16	0.0401 (19)	0.0319 (18)	0.048 (2)	0.0054 (15)	0.0032 (16)	0.0113 (16)
C17	0.056 (3)	0.0281 (18)	0.068 (3)	0.0121 (17)	0.001 (2)	0.0058 (19)
C18	0.062 (3)	0.0242 (17)	0.072 (3)	0.0041 (17)	0.004 (2)	−0.0074 (19)
C19	0.042 (2)	0.0251 (16)	0.048 (2)	−0.0041 (14)	0.0037 (16)	−0.0028 (15)
C20	0.053 (2)	0.040 (2)	0.049 (2)	−0.0090 (18)	−0.0010 (19)	−0.0123 (18)
C21	0.041 (2)	0.050 (2)	0.040 (2)	−0.0082 (17)	−0.0066 (16)	−0.0077 (17)
C22	0.0298 (16)	0.0386 (18)	0.0343 (17)	−0.0006 (13)	−0.0018 (13)	0.0008 (14)
C23	0.0308 (16)	0.0243 (14)	0.0346 (16)	−0.0019 (12)	0.0018 (13)	0.0015 (12)
C24	0.0302 (15)	0.0274 (15)	0.0335 (16)	0.0011 (12)	0.0044 (13)	0.0052 (12)
C25	0.0325 (16)	0.0280 (15)	0.0248 (14)	0.0066 (12)	−0.0026 (12)	−0.0060 (11)
C26	0.0326 (16)	0.0332 (17)	0.0250 (15)	0.0076 (13)	−0.0036 (12)	−0.0022 (13)
C27	0.0254 (14)	0.0275 (15)	0.0287 (15)	0.0053 (11)	−0.0021 (11)	−0.0070 (12)
C28	0.0272 (15)	0.0353 (17)	0.0320 (16)	0.0006 (13)	−0.0024 (12)	−0.0101 (14)

### Geometric parameters (Å, °)

**Table:**

Cu1—O2	1.948 (3)	C4—C12	1.398 (5)
Cu1—O5	1.966 (3)	C4—C5	1.437 (6)
Cu1—N2	2.011 (3)	C5—C6	1.342 (7)
Cu1—N1	2.015 (3)	C5—H5	0.9300
Cu1—O4	2.240 (3)	C6—C7	1.441 (6)
Cu1—Cu2	3.0322 (6)	C6—H6	0.9300
Cu2—O1	1.946 (3)	C7—C11	1.401 (5)
Cu2—O6	1.952 (3)	C7—C8	1.405 (6)
Cu2—N4	2.007 (3)	C8—C9	1.364 (7)
Cu2—N3	2.025 (3)	C8—H8	0.9300
Cu2—O3	2.160 (3)	C9—C10	1.396 (6)
O1—C25	1.258 (5)	C9—H9	0.9300
O2—C25	1.264 (5)	C10—H10	0.9300
O3—H4	0.73 (7)	C11—C12	1.432 (5)
O3—H16	0.70 (6)	C13—C14	1.405 (6)
O4—H24	0.82 (5)	C13—H13	0.9300
O4—H23	0.82 (6)	C14—C15	1.369 (7)
O5—C27	1.259 (4)	C14—H14	0.9300
O6—C27	1.260 (5)	C15—C16	1.415 (6)
O7—N6	1.114 (14)	C15—H15	0.9300
O8—N6	1.099 (14)	C16—C24	1.403 (5)
O9—N6	1.199 (14)	C16—C17	1.443 (7)
O10—N5	1.216 (8)	C17—C18	1.344 (7)
O11—N5	1.231 (8)	C17—H17	0.9300
O12—N5	1.224 (8)	C18—C19	1.436 (6)
O13—H13D	0.70 (7)	C18—H18	0.9300
O13—H13C	0.75 (8)	C19—C23	1.407 (5)
O14—H14B	0.83 (7)	C19—C20	1.411 (6)
O14—H14C	0.82 (8)	C20—C21	1.364 (7)
N1—C1	1.331 (4)	C20—H20	0.9300
N1—C12	1.362 (4)	C21—C22	1.400 (6)
N2—C10	1.326 (5)	C21—H21	0.9300
N2—C11	1.360 (5)	C22—H22	0.9300
N3—C13	1.327 (5)	C23—C24	1.426 (5)
N3—C24	1.358 (5)	C25—C26	1.510 (5)
N4—C22	1.334 (5)	C26—C28^i^	1.510 (5)
N4—C23	1.359 (4)	C26—H26B	0.90 (6)
C1—C2	1.399 (6)	C26—H26A	0.89 (5)
C1—H1	0.9300	C27—C28	1.513 (4)
C2—C3	1.371 (7)	C28—C26^i^	1.510 (5)
C2—H2	0.9300	C28—H28B	0.91 (6)
C3—C4	1.406 (6)	C28—H28A	0.90 (7)
C3—H3	0.9300		
			
O2—Cu1—O5	90.69 (12)	C5—C6—C7	121.6 (4)
O2—Cu1—N2	91.42 (12)	C5—C6—H6	119.2
O5—Cu1—N2	175.95 (12)	C7—C6—H6	119.2
O2—Cu1—N1	164.53 (12)	C11—C7—C8	117.1 (4)
O5—Cu1—N1	95.00 (12)	C11—C7—C6	118.1 (4)
N2—Cu1—N1	82.10 (12)	C8—C7—C6	124.8 (4)
O2—Cu1—O4	102.84 (14)	C9—C8—C7	119.4 (4)
O5—Cu1—O4	95.40 (14)	C9—C8—H8	120.3
N2—Cu1—O4	87.49 (14)	C7—C8—H8	120.3
N1—Cu1—O4	90.95 (14)	C8—C9—C10	120.0 (4)
O2—Cu1—Cu2	83.04 (9)	C8—C9—H9	120.0
O5—Cu1—Cu2	79.10 (9)	C10—C9—H9	120.0
N2—Cu1—Cu2	97.73 (9)	N2—C10—C9	122.3 (4)
N1—Cu1—Cu2	83.93 (8)	N2—C10—H10	118.9
O4—Cu1—Cu2	172.09 (12)	C9—C10—H10	118.9
O1—Cu2—O6	91.46 (13)	N2—C11—C7	123.0 (3)
O1—Cu2—N4	93.81 (12)	N2—C11—C12	116.7 (3)
O6—Cu2—N4	164.98 (13)	C7—C11—C12	120.2 (3)
O1—Cu2—N3	173.84 (13)	N1—C12—C4	123.5 (3)
O6—Cu2—N3	91.23 (13)	N1—C12—C11	116.2 (3)
N4—Cu2—N3	82.25 (12)	C4—C12—C11	120.3 (3)
O1—Cu2—O3	93.07 (14)	N3—C13—C14	121.4 (4)
O6—Cu2—O3	97.50 (14)	N3—C13—H13	119.3
N4—Cu2—O3	96.25 (14)	C14—C13—H13	119.3
N3—Cu2—O3	92.07 (14)	C15—C14—C13	120.3 (4)
O1—Cu2—Cu1	72.40 (9)	C15—C14—H14	119.9
O6—Cu2—Cu1	77.09 (9)	C13—C14—H14	119.9
N4—Cu2—Cu1	91.12 (9)	C14—C15—C16	119.1 (4)
N3—Cu2—Cu1	102.83 (9)	C14—C15—H15	120.5
O3—Cu2—Cu1	164.16 (11)	C16—C15—H15	120.5
C25—O1—Cu2	134.9 (3)	C24—C16—C15	117.2 (4)
C25—O2—Cu1	121.1 (2)	C24—C16—C17	118.4 (4)
Cu2—O3—H4	122 (5)	C15—C16—C17	124.4 (4)
Cu2—O3—H16	125 (5)	C18—C17—C16	121.5 (4)
H4—O3—H16	110 (7)	C18—C17—H17	119.2
Cu1—O4—H24	144 (7)	C16—C17—H17	119.2
Cu1—O4—H23	118 (5)	C17—C18—C19	121.0 (4)
H24—O4—H23	98 (7)	C17—C18—H18	119.5
C27—O5—Cu1	126.8 (2)	C19—C18—H18	119.5
C27—O6—Cu2	130.2 (2)	C23—C19—C20	116.8 (4)
H13D—O13—H13C	97 (7)	C23—C19—C18	118.7 (4)
H14B—O14—H14C	88 (8)	C20—C19—C18	124.4 (4)
C1—N1—C12	117.8 (3)	C21—C20—C19	119.6 (4)
C1—N1—Cu1	129.7 (3)	C21—C20—H20	120.2
C12—N1—Cu1	112.5 (2)	C19—C20—H20	120.2
C10—N2—C11	118.2 (3)	C20—C21—C22	120.0 (4)
C10—N2—Cu1	129.3 (3)	C20—C21—H21	120.0
C11—N2—Cu1	112.4 (2)	C22—C21—H21	120.0
C13—N3—C24	119.2 (3)	N4—C22—C21	122.1 (4)
C13—N3—Cu2	129.1 (3)	N4—C22—H22	119.0
C24—N3—Cu2	111.7 (2)	C21—C22—H22	119.0
C22—N4—C23	118.2 (3)	N4—C23—C19	123.3 (3)
C22—N4—Cu2	129.3 (3)	N4—C23—C24	116.6 (3)
C23—N4—Cu2	112.4 (2)	C19—C23—C24	120.1 (3)
O10—N5—O12	122.5 (7)	N3—C24—C16	122.8 (3)
O10—N5—O11	117.0 (6)	N3—C24—C23	117.0 (3)
O12—N5—O11	120.4 (6)	C16—C24—C23	120.1 (3)
O8—N6—O7	119.1 (16)	O1—C25—O2	125.4 (3)
O8—N6—O9	112.7 (14)	O1—C25—C26	116.4 (3)
O7—N6—O9	127.8 (14)	O2—C25—C26	118.1 (3)
N1—C1—C2	122.4 (4)	C25—C26—C28^i^	113.2 (3)
N1—C1—H1	118.8	C25—C26—H26B	113 (4)
C2—C1—H1	118.8	C28^i^—C26—H26B	110 (4)
C3—C2—C1	119.9 (4)	C25—C26—H26A	106 (3)
C3—C2—H2	120.1	C28^i^—C26—H26A	114 (3)
C1—C2—H2	120.1	H26B—C26—H26A	99 (5)
C2—C3—C4	119.2 (4)	O5—C27—O6	125.5 (3)
C2—C3—H3	120.4	O5—C27—C28	117.9 (3)
C4—C3—H3	120.4	O6—C27—C28	116.6 (3)
C12—C4—C3	117.2 (4)	C26^i^—C28—C27	114.8 (3)
C12—C4—C5	118.5 (4)	C26^i^—C28—H28B	114 (4)
C3—C4—C5	124.3 (4)	C27—C28—H28B	103 (4)
C6—C5—C4	121.3 (4)	C26^i^—C28—H28A	116 (4)
C6—C5—H5	119.4	C27—C28—H28A	97 (4)
C4—C5—H5	119.4	H28B—C28—H28A	110 (5)

Symmetry codes: (i) −*x*+1, −*y*, −*z*.

### Hydrogen-bond geometry (Å, °)

**Table:**

*D*—H···*A*	*D*—H	H···*A*	*D*···*A*	*D*—H···*A*
O3—H4···O14	0.73 (7)	1.99 (7)	2.707 (7)	169 (7)
O3—H16···O13	0.70 (6)	2.07 (6)	2.772 (6)	177 (9)
O4—H23···O12	0.82 (6)	2.27 (6)	3.015 (7)	153 (5)
O4—H24···O10^ii^	0.82 (5)	2.03 (6)	2.816 (6)	161 (8)
O13—H13C···O5^i^	0.77 (7)	2.24 (7)	3.000 (5)	167 (7)
O13—H13D···O10^iii^	0.71 (7)	2.23 (7)	2.899 (7)	159 (8)
O14—H14B···O9^iv^	0.83 (7)	2.15 (6)	2.846 (13)	142 (6)
O14—H14C···O7^v^	0.82 (9)	2.10 (9)	2.874 (14)	158 (10)

Symmetry codes:  (ii) −*x*, −*y*, −*z*+1; (i) −*x*+1, −*y*, −*z*; (iii) *x*+1, *y*, *z*−1; (iv) *x*+1, −*y*+1/2, *z*−1/2; (v) *x*+1, *y*, *z*.
